# Inhibition of post-transcriptional steps in ribosome biogenesis confers cytoprotection against chemotherapeutic agents in a p53-dependent manner

**DOI:** 10.1038/s41598-017-09002-w

**Published:** 2017-08-22

**Authors:** Russell T. Sapio, Anastasiya N. Nezdyur, Matthew Krevetski, Leonid Anikin, Vincent J. Manna, Natalie Minkovsky, Dimitri G. Pestov

**Affiliations:** 1 Department of Cell Biology and Neuroscience, Rowan University School of Osteopathic Medicine, Stratford, NJ 08084 USA; 2 Graduate School of Biomedical Sciences, Rowan University School of Osteopathic Medicine, Stratford, NJ 08084 USA; 3 Department of Chemistry and Biochemistry, Rowan University, Glassboro, NJ 08028 USA; 4 Department of Biological Sciences, Rowan University, Glassboro, NJ 08028 USA

## Abstract

The p53-mediated nucleolar stress response associated with inhibition of ribosomal RNA transcription was previously shown to potentiate killing of tumor cells. Here, we asked whether targeting of ribosome biogenesis can be used as the basis for selective p53-dependent cytoprotection of nonmalignant cells. Temporary functional inactivation of the 60S ribosome assembly factor Bop1 in a 3T3 cell model markedly increased cell recovery after exposure to camptothecin or methotrexate. This was due, at least in part, to reversible pausing of the cell cycle preventing S phase associated DNA damage. Similar cytoprotective effects were observed after transient shRNA-mediated silencing of Rps19, but not several other tested ribosomal proteins, indicating distinct cellular responses to the inhibition of different steps in ribosome biogenesis. By temporarily inactivating Bop1 function, we further demonstrate selective killing of p53-deficient cells with camptothecin while sparing isogenic p53-positive cells. Thus, combining cytotoxic treatments with inhibition of select post-transcriptional steps of ribosome biogenesis holds potential for therapeutic targeting of cells that have lost p53.

## Introduction

The curative potential of chemotherapeutic agents is limited by their toxicity toward normal cells. One approach to reducing side effects of S- or M-phase-specific anticancer drugs is to induce a reversible cell cycle arrest in the host’s normal cells during treatment^1–4^. This cytoprotection strategy, also known as cyclotherapy, was shown to improve chemotherapeutic drug efficacy in cell models^5–8^, but finding suitable ways to selectively halt the cell cycle in normal cells has remained challenging. The tumor suppressor p53, commonly lost or mutated in human cancers, has been a primary candidate for the selective cytoprotection of nonmalignant cells^7, 9–12^. For example, activation of wild-type p53 with the MDM2 antagonist nutlin-3 was found to increase tolerance of cells to a variety of cytotoxic treatments^7, 10, 11^. However, p53 activation can also be harmful for vulnerable tissues^13^, making it critical that levels and duration of its activity are carefully controlled in any p53-based therapy^14^. Identifying suitable cellular targets for activating p53 in a controllable manner would be necessary to fully exploit cyclotherapy as a cancer treatment option.

Changes in gene expression mediated by p53 are integral to cellular responses to many different kinds of stress, including those that do not normally lead to cell lethality but induce metabolic reprogramming and cell cycle arrest^15–17^. In principle, invoking such stress conditions could activate a program that facilitates cell survival. Perturbation of ribosome biogenesis in the nucleolus (often referred to as nucleolar, or ribosomal stress) was shown to activate nongenotoxic p53-mediated responses in cells, with outcomes dependent on cell type, preceding rate of ribosome synthesis and nature of the perturbation^18–25^. Ribosome biogenesis is an essential, multistep process that requires hundreds of components including ribosomal RNA (rRNAs), small nucleolar RNAs, ribosomal proteins (r-proteins) and auxiliary assembly factors to synthesize new ribosomal subunits^26–28^. One mechanism by which cells sense impairment of ribosome biogenesis is through binding of unassembled r-proteins and 5S rRNA to MDM2, resulting in the inhibition of the MDM2 ubiquitin ligase activity toward p53 and activation of p53 targets (reviewed in^29, 30^). Additional aspects of the cellular response to impaired ribosome biogenesis continue to emerge, indicating contribution of multiple signaling pathways^31–40^.

In cancer therapy, targeting the nucleolus with inhibitors of Pol I-driven rRNA transcription has been used to kill tumor cells^41, 42^. For example, the selective small-molecule inhibitor of Pol I transcription CX-5461^43^ was found to improve the clinical effectiveness in treatments of lymphoma and several other human malignancies^44, 45^. In contrast, only few studies have explored the potential therapeutic utility of the cytostatic responses triggered by nucleolar stress. The DNA-binding drug actinomycin D was previously shown to inhibit elongation of growing rRNA transcripts by Pol I at nanomolar concentrations^46, 47^ and cause stabilization of p53, attributed to nucleolar disruption^48–50^. The same range of actinomycin D concentrations was reported to protect cells from anti-mitotic drugs in a p53-dependent manner^6, 8, 51^. However, actinomycin D has a narrow therapeutic window as a chemoprotectant, with higher doses rapidly becoming cytotoxic^6, 51^.

In this study, we tested the idea that inhibition of post-transcriptional ribosome assembly steps might provide a useful way of protecting p53-proficient cells against chemotherapeutic drugs. Compared with Pol I transcription, ribosome assembly is significantly more complex^27, 28^, offering a wide diversity of targets that could be potentially exploited for activating p53 in a nongenotoxic manner. However, it remains to be established whether transiently inhibiting post-transcriptional ribosome biogenesis steps can elicit prosurvival responses, suitable for modulating cell sensitivity to chemotherapeutic agents. Using a model cell system, we demonstrate here the enhancement of cell resistance to cell cycle-specific cytotoxic drugs in a p53-dependent manner by transient interference with a ribosome assembly factor and an r-protein. As a result, selective killing of p53-deficient cells while protecting p53-positive cells can be achieved. These findings suggest that targeting specific post-transcriptional ribosome assembly steps can be used in combination with existing cytotoxic drugs to design improved therapeutic interventions in cancer.

## Results

### Targeting ribosome biogenesis via Bop1 enhances cell chemoresistance

**Figure 1:**
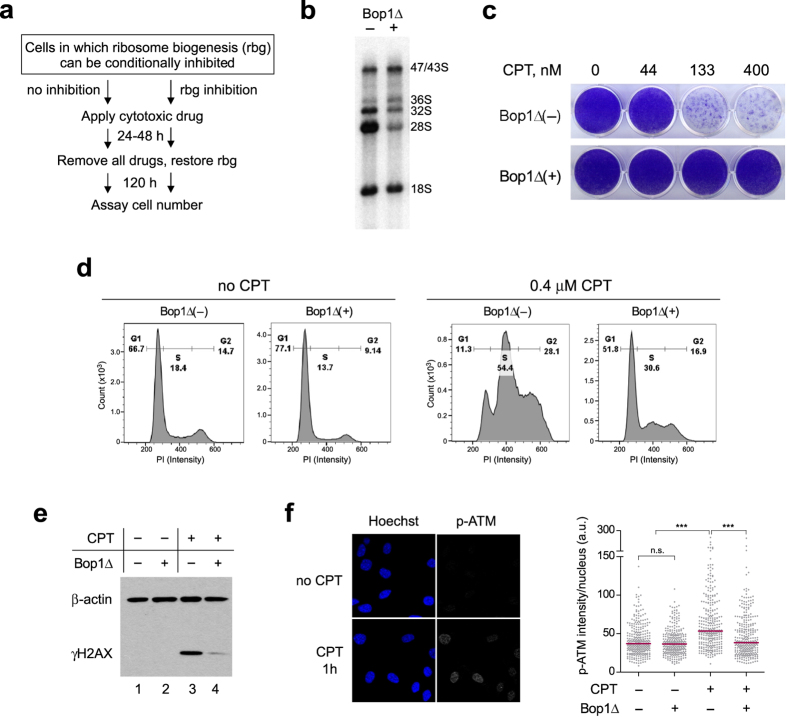
Expression of the dominant-negative Bop1 mutant, Bop1Δ, confers protection against camptothecin (CPT). (**a**) Experimental protocol for testing cytoprotective effects of the inhibition of ribosome biogenesis. (**b**) ^32^P labeling of rRNA shows reduced levels of the newly synthesized 28S rRNA and altered levels of 36S, 32S pre-rRNAs in D411 cells after induction of Bop1Δ for 24 h (+) as compared to uninduced cells (–). (**c**) Expression of Bop1Δ in D411 cells was either induced (+) for 24 h with 1 mM IPTG or not induced (–), and equal numbers of cells were then treated for 24 h with the indicated concentrations of CPT. Cells were stained with crystal violet 5 days after treatment. No IPTG was present in medium during the recovery period. (**d**) Representative DNA profiles of D411 cells that were untreated or treated with 0.4 μM CPT for 24 h. Prior to drug treatment, cells were either incubated or not with IPTG for 24 h to induce Bop1Δ. Following drug treatment, cells were harvested, permeabilized and their cell cycle profiles determined by staining with propidium iodide (PI). (**e**) Bop1Δ expression attenuates γH2AX levels induced by CPT. D411 cells were either incubated or not with IPTG for 24 h and exposed to 0.4 μM CPT for 1 h. γH2AX and β-actin (loading control) were detected by immunoblotting. The experiment was repeated four times with similar results. The original immunoblot images are shown in Supplementary Figure S7. (f) Representative images illustrating increased ATM phosphorylation in the nuclei of cells treated with 0.4 μM CPT for 1 h. Immunofluorescence analysis was done with p-ATM(Ser1981) monoclonal antibodies. Quantification of the antibody signal shows its significant reduction in Bop1Δ-expressing cells (*n* = 300 nuclei per group; ***P < 0.0001, Student’s two-tailed *t*-test; horizontal line denotes the median).

An effective cytoprotection of nonmalignant cells against toxic drugs that target cycling tumor cells requires a sustained interruption of the cell cycle for the duration of the treatment necessary to kill tumor cells. This cell cycle arrest should also be reversible and produce no additional cellular damage in wild-type cells. To investigate whether short-term targeting of specific steps in ribosome biogenesis could fulfill these criteria, we performed cell survival assays in which we split cells into two treatment groups, temporarily disrupted ribosome biogenesis in one of the groups, and treated all cells with a cytotoxic chemical (Fig. 1a). Cells were then maintained for 5 days in drug-free conditions to distinguish cells that successfully recovered from the treatment and resumed proliferation from those that were damaged and irreversibly arrested.

We began to examine the effects of targeting post-transcriptional ribosome biogenesis steps by reversibly inhibiting function of the conserved 60S subunit ribosome assembly factor Bop1^52, 53^. Previous studies have shown that conditional expression of the dominant-negative Bop1 mutant Bop1Δ in 3T3 cells stalls 60S subunit maturation and induces p53-dependent G1 arrest, both of which can be reversed when normal Bop1 function in cells is restored^19^. Analysis of radiolabeled rRNA in 3T3 D411 cells harboring IPTG-inducible Bop1Δ showed reduced synthesis of 28S rRNA and altered levels of its precursors (Fig. 1b), indicative of impaired 60S subunit maturation^52, 53^. To test whether interfering with Bop1 function alters cell chemoresistance, we challenged the cells with a range of doses of camptothecin (CPT), a topoisomerase I inhibitor that has a strong cytotoxic activity during DNA replication^54–56^, and evaluated clonogenic cell survival. As shown in Fig. 1c, perturbing Bop1 function via transient expression of its dominant-negative mutant effectively protected cells from lethal doses of CPT. When Bop1Δ expression was shut down again, both ribosome biogenesis and cell proliferation were restored, and this was accompanied by loss of cytoprotection (Supplementary Fig. S1).

### Targeting Bop1 function reduces CPT-induced DNA damage by preventing S phase entry

**Figure 2:**
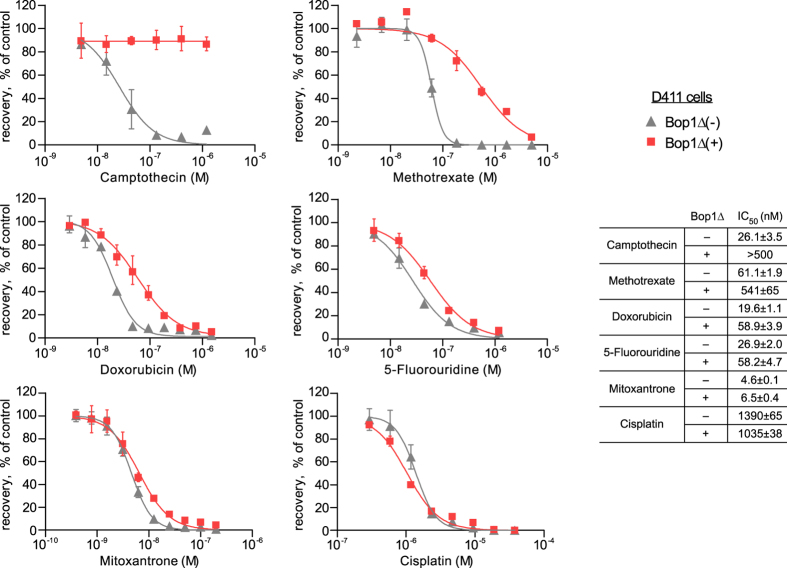
Bop1Δ expression increases cell survival in treatments with chemotherapeutic drugs. Bop1Δ was induced for 24 h with IPTG or not induced, and cells were treated with various concentrations of the indicated drugs for 24 h, except MTX treatment, which was 48 h. After recovery for 120 h in drug-free medium, viable cells were quantified using alamarBlue assays as detailed in Methods. Data in each graph are normalized to the no-treatment control and show mean and s.d. values in triplicate samples; where error bars are not visible, they are smaller than the symbol size. The tabulated IC_50_ values are presented as mean ± s.e.m.

CPT causes DNA damage in cells by stabilizing topoisomerase I-DNA complexes and preventing DNA strand religation^55^. The resulting ternary complexes arrest progression of replication forks and transcription bubbles, causing double-stranded DNA breaks and loss of viability^54, 56^. We used flow cytometry to examine cellular DNA content in cells after CPT treatment, both with and without prior induction of Bop1Δ. In cells where Bop1Δ was not induced, 0.4 μM CPT treatment caused an abnormal accumulation of cells with S phase DNA content (Fig. 1d), consistent with the replicative stress caused by CPT. This accumulation was greatly decreased if Bop1Δ was induced prior to CPT treatment (Fig. 1d, right panel), suggesting that the G1 arrest induced by Bop1Δ expression^19^ blocks cells from initiating DNA replication in the presence of the drug. Immunoblotting analysis of cycling cells exposed to CPT showed increased levels of phosphorylated γH2AX, a marker of double-stranded DNA breaks (Fig. 1e). Induction of the Bop1Δ mutant prior to CPT treatment caused a clear reduction in γH2AX levels (Fig. 1e, lanes 3 and 4), consistent with the protective effect of Bop1Δ expression. Notably, expression of Bop1Δ by itself did not increase γH2AX levels, suggesting that a temporary inhibition of ribosome biogenesis in cells through this mutant was not genotoxic. To further confirm that Bop1Δ expression reduces the extent of CPT-induced DNA damage, we examined phosphorylation of ATM, activated by DNA double-strand breaks. Immunofluorescence analysis (Fig. 1f) showed that a short CPT treatment significantly increased the number of cell nuclei with an intense p-ATM signal, consistent with widespread DNA damage in these cells, but this increase was diminished when Bop1Δ was induced prior to drug treatment. We next examined dose responses of cells expressing the Bop1Δ mutant to different cytotoxic drugs (Fig. 2). We observed robust protection against CPT and methotrexate (MTX), two drugs that exert damage primarily during DNA replication. Induction of Bop1Δ was less effective with doxorubicin and 5-fluorouridine, whose cytotoxicity is partially S-phase dependent^57, 58^, and had minimal effects on cell survival with mitoxantrone and cisplatin, which target cells throughout the cell cycle^56^, consistent with the idea that blocking S phase entry was important for Bop1Δ-induced chemoprotection.

### Loss of p53 abolishes the cytoprotective properties of Bop1Δ

**Figure 3:**
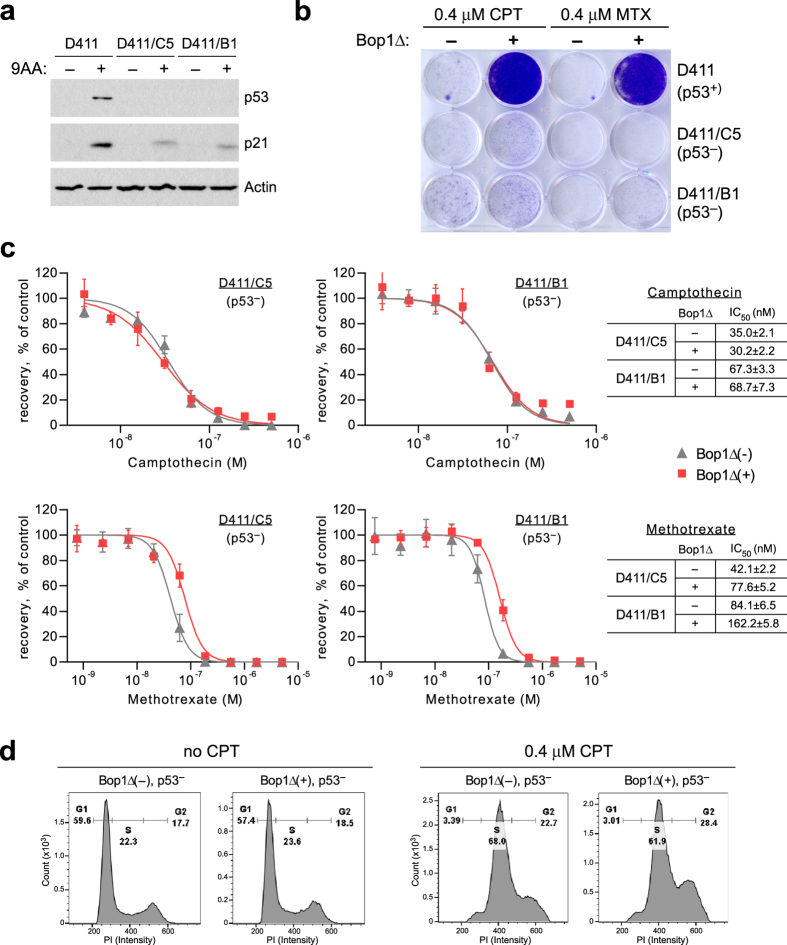
p53 is necessary for nucleolar stress-induced cytoprotection. (**a**) Verification of the p53-deficient status of Bop1Δ-inducible cell lines D411/C5 and D411/B1. To upregulate p53 protein levels, cells were treated for 6 h with 10 μM 9-aminoacridine (9AA), a strong activator of p53^77^. Representative immunoblots of p53 and p21 levels are shown in comparison with the parental D411 cell line; β-actin was used as a loading control. Full-size immunoblot images are shown in Supplementary Figure S7. (**b**) p53-deficient cells are sensitive to CPT and MTX regardless of the induction of Bop1Δ. D411 and its p53-null derivatives were stained with crystal violet after treatment with CPT for 24 h or MTX for 48 h as detailed in Fig. 1c. (**c**) Bop1Δ induction has little to no effect on the dose response to CPT and MTX in the p53-deficient cell lines. The assay was performed as in Fig. 2. (**d**) Representative DNA profiles of the D411/C5 cells show that S-phase accumulation caused by 0.4 μM CPT is not affected by Bop1Δ expression on the p53-null background. Cell cycle analysis was performed as in Fig. 1d.

Previously, we have shown that inducing perturbation in ribosome biogenesis via dominant-negative Bop1 mutations activates the p53 pathway^19^. To determine whether p53 is required for the chemoresistance phenotype associated with Bop1Δ expression, we generated Bop1Δ-inducible cells lacking p53 by using CRISPR/Cas9. In two independently obtained clonal derivatives of D411 cells, disruption of all p53 alleles was confirmed by sequencing their PCR-amplified p53 loci (Supplementary Fig. S2). These cells lacked detectable p53 protein and had diminished expression of the p53-regulated p21 protein in western blot analysis (Fig. 3a). We also verified that Bop1Δ induction in the generated p53-null cells still led to defects in ribosome biogenesis by northern blot analysis of rRNA precursors (Supplementary Fig. S3). We next treated two generated p53-null cell lines, D411/C5 and D411/B1, with CPT and assessed their survival in comparison with the parental D411 cells. In contrast to the parental p53-positive cell line, induction of Bop1Δ in p53-deficient cells failed to provide resistance to CPT and only marginally increased resistance to MTX (Fig. 3b,c). Flow cytometry analysis of the cell cycle showed that unlike the parental cells, the p53-null cells failed to arrest in G1 and accumulated in S phase, suggestive of CPT-induced replication damage (Fig. 3d, Supplementary Fig. S4, compare with Fig. 1d). Based on these data, we conclude that p53 is required for Bop1Δ-induced cytoprotection.

### Targeting ribosomal protein Rps19 confers cytoprotection

**Figure 4:**
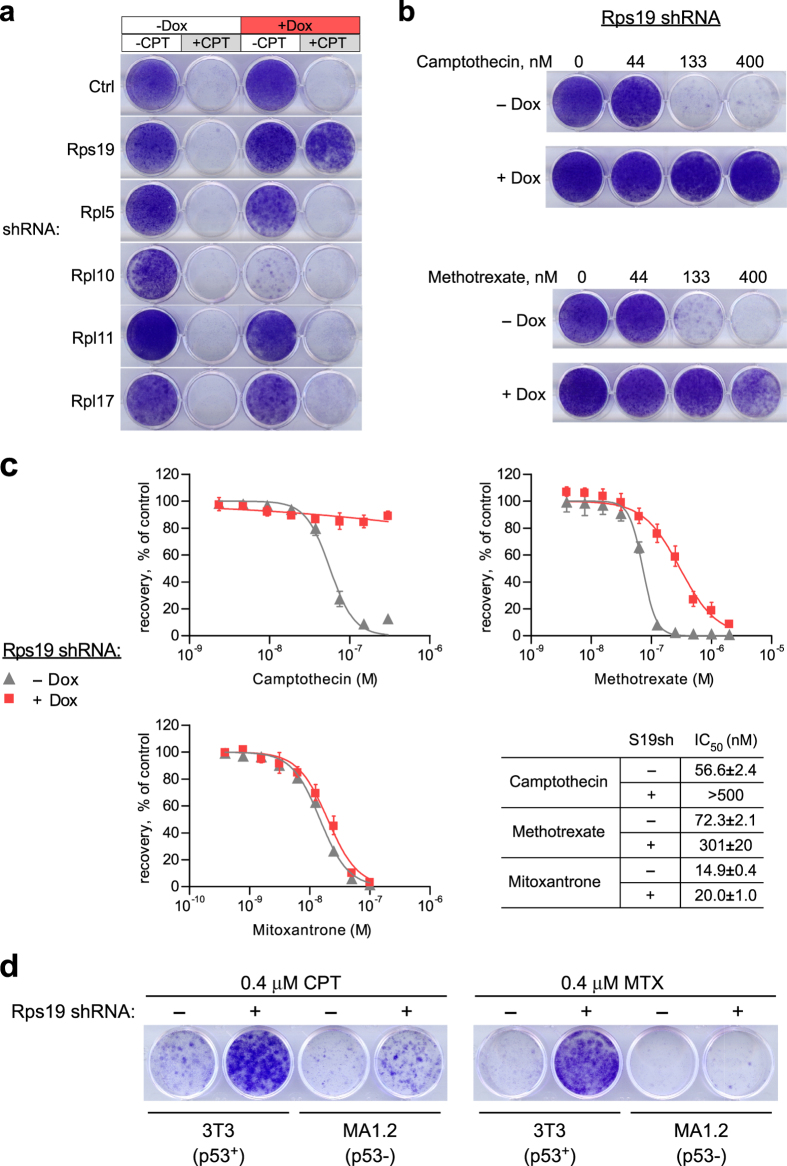
Transient depletion of r-proteins via Dox-inducible shRNA identifies Rps19 as an inducer of cytoprotection against CPT and MTX. (**a**) The indicated shRNAs were either induced with Dox for 48 h or not induced, cells were treated with 0 or 0.4 μM CPT for 24 h and the surviving viable cells were stained with crystal violet after a 5-day recovery in Dox-free medium. (**b**) Rps19 shRNA was induced for 48 h with Dox or not induced, cells were treated with the indicated concentrations of CPT for 24 h or MTX for 48 h and stained as above. (**c**) Dox induction of Rps19 shRNA confers protection against CPT and MTX, but not mitoxantrone. Cells were induced for 48 h with Dox or not induced and then treated with various concentrations of CPT and mitoxantrone for 24 h or with MTX for 48 h. Results were analyzed as in Fig. 2. (**d**) p53 is necessary for cytoprotective effects of Rps19 depletion. 3T3 cells and its p53-null derivative line MA1.2 were transfected with Dox-inducible Rps19 shRNA and analyzed as in Fig. 3b.

The chemoprotective properties of the inhibition of Bop1 function raised the question of whether other ribosome biogenesis targets might elicit similar effects. Previous studies have shown that depletion of individual r-proteins can disrupt different steps of ribosome assembly and activate p53^27, 59^. We used doxycycline-inducible expression of shRNAs to knock down several representative r-proteins with previously characterized roles in the ribosome biogenesis pathway and impact on p53 homeostasis. We chose to test Rpl17 (also known as uL22) and Rps19 (eS19), rated as strong and intermediate p53 impactors, respectively^59^. We also tested knockdowns of Rpl5 (uL18) and Rpl11 (uL5), which affect 60S subunit biogenesis, but at the same time impair the p53 response^49, 60, 61^. Lastly, Rpl10 (uL16) is required for normal translation and cell proliferation^62^, but this r-protein is assembled into ribosomes in the cytoplasm, playing no direct role in their nucleolar maturation^27^. We induced expression of the shRNAs with doxycycline for 2 days, treated cells with CPT, and examined clonogenic cell survival. Knockdown efficiencies of the shRNAs were monitored by qRT-PCR (Supplementary Fig. S5). Among the tested r-protein targets, we found that depletion of Rps19, an r-protein required for 40S subunit formation^27, 63–65^, significantly increased survival of cells in CPT (Fig. 4a). As expected, we did not see improved cell survival with shRNAs targeting Rpl5 or Rpl11, two r-proteins essential for p53 activation by nucleolar stress^30, 49, 60, 61^. Depletion of the cytoplasmic r-protein Rpl10 was not only ineffective for protection against CPT, but also detrimental for cell viability on its own (Fig. 4a). Interestingly, depletion of Rpl17, which strongly inhibits 60S biogenesis and cell proliferation^62^, failed to provide efficient cytoprotection for a different reason. Microscopy analysis showed that the majority of Rpl17-depleted cells displayed senescent-like morphology after CPT treatment and appeared irreversibly arrested, a phenotype not observed with either Bop1Δ or Rps19 depletion. Collectively, these observations show that chemoprotection is not universally associated with deficiencies in ribosome biogenesis, but is target-specific.

We next evaluated the effects of the conditional knockdown of Rps19 in more detail. We found that targeting Rps19 increased cell survival upon exposure to CPT, MTX, but not to mitoxantrone (Fig. 4b,c), closely resembling the effects of inhibiting Bop1 function (see Fig. 2). To determine whether targeting Rps19 confers cytoprotection in a p53-dependent manner, we generated independent p53-null derivatives of 3T3 cells using CRISPR/Cas9 editing (Supplementary Fig. S2), and transfected these cells with a Dox-inducible Rps19 shRNA construct. Transient downregulation of Rps19 expression showed increased survival after treatment with a lethal dose of CPT or MTX in cells with intact p53, but failed to protect p53-null cells (Fig. 4d), indicating that Rps19-associated chemoprotection depended on p53 status. Cell cycle analysis confirmed that p53-null cells were unable to arrest in G1 after Rps19 shRNA expression and displayed abnormal accumulation in S phase when treated with 0.4 μM CPT (Supplementary Fig. S6), similar to the effects observed above with Bop1Δ. The similarity of the phenotypes obtained with Bop1 and Rps19 suggests that p53-mediated chemoprotection can be achieved with targeted disruptions in either the 60S or 40S branch of the ribosome biogenesis pathway.

### The synergistic action of Bop1Δ and CPT allows selective killing of p53-negative cells

**Figure 5:**
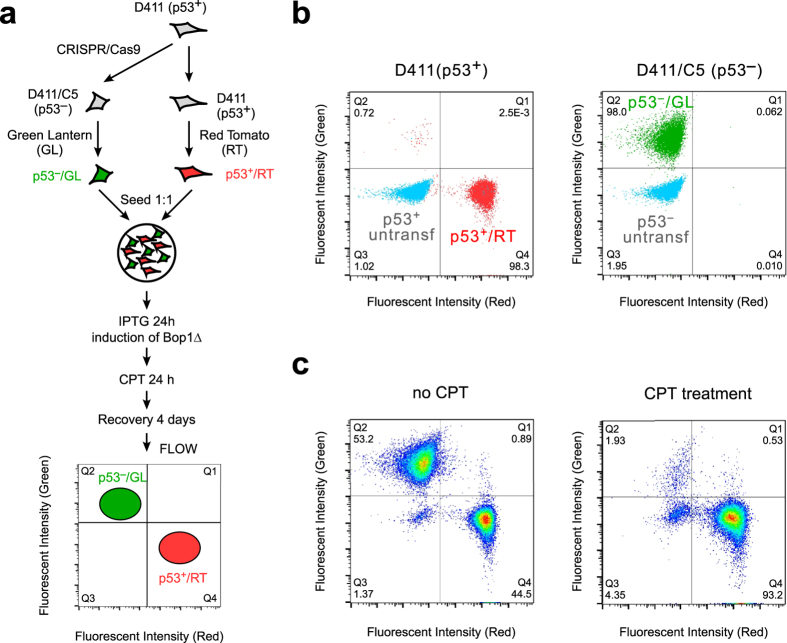
The combination of nucleolar stress and CPT leads to selective depletion of p53-negative cells from a mixed cell population. (**a**) A cell model to test the combination of a transient nucleolar stress with the CPT treatment in isogenic p53+ and p53- cells. (**b**) Flow cytometry plots of D411 (p53+) cells and D411/C5 (p53-) cells transfected with the red (RT) and green (GL) fluorescent markers, respectively. (**c**) Representative flow cytometry analysis of the mixed fluorescently labeled p53+ and p53- cells after induction of Bop1Δ and 0.4 μM CPT treatment indicates increased survival of RT-labeled p53+ cells relative to GL-labeled p53- cells. In the absence of Bop1Δ induction, cells are killed by this concentration of CPT regardless of p53 status (see Fig. 3b).

Given the p53 dependency of the chemoprotection phenotype observed above, we sought to test the idea that inducing nucleolar stress in conjuction with cytotoxic treatment might be used for the selective killing of cells that have lost p53. To this end, we generated fluorescently labeled wild-type p53, Bop1Δ-inducible D411 cells and their p53-deficient D411/C5 derivatives using the Red Tomato (RT) and Green Lantern (GL) fluorescent proteins, respectively. This enabled tracking of each cell type in a mixed population using flow cytometry (Fig. 5a). The RT-labeled (p53 + ) and GL-labeled (p53-) cells (Fig. 5b) were mixed in a 1:1 ratio, incubated with IPTG for 24 h to induce expression of the Bop1 mutant, treated with CPT for 24 h, and analyzed by flow cytometry after 4-day recovery in drug-free conditions. In a control mixed culture that was not treated with CPT, the percentages of the p53+/RT+ and p53-/GL+ cells were at this point 45% and 53%, respectively, indicating comparable growth rates of both types of cells (Fig. 5c, left). Analysis of cells treated with CPT, by contrast, revealed a strong reduction in the number of p53-/GL+ cells, with their fraction decreasing to < 2% (Fig. 5c, right). Thus, protection of p53-proficient cells by Bop1Δ expression can effectively direct CPT cytotoxicity against p53-negative cells, leading to their selective depletion in a mixed cell population.

## Discussion

The experiments reported here indicate that disruptions in the post-transcriptional ribosome assembly pathway are capable of greatly increasing the resistance of p53-positive cells to S phase-specific chemotherapeutic drugs. These results suggest a new approach for selective, p53-dependent chemoprotection of cells by targeted inhibition of ribosome assembly steps. This may help decrease side effects and enhance the efficacy of anticancer drugs.

In our study, we tested genetic inhibition of several factors required for ribosome biosynthesis to assess their ability to induce a chemoprotective response. Conditional inhibition of the function of the 60S subunit assembly factor Bop1 reduced cytotoxic DNA damage upon exposure of cells to high doses of CPT and MTX and profoundly increased cell survival. Downregulation of Rps19, which affects 40S maturation, also provided robust cytoprotection against CPT and MTX. This behavior was not observed with several other tested r-proteins in the same cell model. The difference in these outcomes may be due to several reasons. The previously reported variations in the extent of p53 activation between knockdowns of individual r-proteins^59^ might be one contributing factor. It is also possible that defects in different ribosome biogenesis steps may generate distinct combinations of stress inputs for the nucleolar surveillance network. Indeed, recent studies have shown that monitoring nucleolar function involves a spectrum of mechanisms that may work in parallel to p53-mediated signaling or influence its outcome^32–40^.

For our proof-of-concept experiments reported here, we used 3T3 cells in which interference with ribosome biogenesis halts cell cycle progression^19^. Cytostatic responses to nucleolar stress have been observed in other cell types^18, 21, 23, 66^. To exploit nucleolar stress as a therapeutic cytoprotection strategy, more studies will be needed to better understand the effects of short-term disruption of ribosome biogenesis in cells and tissues *in vivo*. Previous studies in human patients and animal models focused mainly on congenital genetic defects that affect ribosome biogenesis over the lifetime of an organism^32, 36, 67^. Persistent nucleolar stress of this type can lead to increased levels of both proliferative arrest and apoptosis in the bone marrow, eventually leading to the exhaustion of hematopoietic stem cells^23, 24^. At least in part this phenotype seems to reflect the general hypersensitivity of the hematopoietic system to p53 activation, a known obstacle in therapeutic applications that activate p53 signaling^13^. It is worth noting that recent findings point to the underlying plasticity in the cellular response to nucleolar stress. For example, it was reported that cell ribosome synthesis rate determines whether apoptosis or cell cycle arrest is induced by inhibition of ribosome biogenesis^25^. Another recent study has shown that targeting specific innate immune response components can ameliorate the erythropoietic defect associated with Rps14 haploinsufficiency in mice^38^. Thus, it may be possible to design combinatorial treatments that employ the beneficial chemoprotective properties of short-term nucleolar stress while minimizing potential side effects in sensitive tissues.

Considering the target dependence of the cytoprotection observed in our model system, development of inhibitors of specific post-transcriptional ribosome biogenesis steps may be necessary to harness the cytoprotection potential of nucleolar stress for *in vivo* applications. Inhibitors of Pol I-driven transcription of rRNA are currently the most commonly used drugs to perturb nucleolar function^43, 44, 50, 68^. A variety of antimetabolic drugs used in cancer therapy are also known to disrupt ribosome biogenesis^69, 70^, although their associated genotoxic effects may limit their usability as selective inducers of nucleolar stress. Our results identify Bop1 and Rps19 as two candidate targets for designing small molecules to induce a cytoprotective nucleolar stress response and reduce damage to normal tissues from anticancer drugs such as CPT or MTX. Bop1 functions in pre-60S ribosome maturation as a component of the heterotrimeric PeBoW complex^71^. Designing pharmacological agents to disrupt interactions within this complex might provide one potential approach to selectively stall ribosome biogenesis. Ribosome assembly requires several hundred proteins^26^ that may reveal other promising targets for modulating cellular chemoresistance.

## Methods

### Reagents and antibodies

CPT and cisplatin were obtained from Sigma; MTX, mitoxantrone, doxorubucin from Enzo Life Sciences, 5-fluorouridine from Acros Organics. The following antibodies were used: anti-p53 1:1,000 (Thermo Scientific, clone PAB240), anti-p21 1:1,000 (Santa Cruz, c-19), anti-γH2AX 1:2,500 (Millipore, JBW301), anti-phospho-ATM Ser1981 1:400 (Invitrogen, 10H11), anti-β-actin 1:10,000 (MP Biomedicals, C4).

### Cell lines and culture conditions

The derivation of the NIH 3T3-based LAP3 cell line for IPTG-inducible expression and its Bop1Δ-expressing clones have been described previously^53^. The D411 line was previously referred to as clone Bop1Δ/6^19^. For conditional knockdowns in 3T3 cells, shRNA sequences were recloned from pGIPZ library constructs (Dharmacon) into the doxycycline-inducible pIVRE vector^72^ and stably transfected into cells using calcium phosphate followed by puromycin selection. Single-cell clones used for drug sensitivity tests displayed > 65% reduction in target mRNA level after doxycycline induction, as determined by qRT-PCR^72^. All cells were maintained in DMEM supplemented with 10% calf serum (HyClone) and 1% penicillin-streptomycin. Cells were regularly monitored for mycoplasma using a PCR detection kit from ATCC. To induce transfected constructs, cells were incubated with 1 mM IPTG (Thermo Fisher AM9462) for 24 h or 2 μg/ml Dox (Frontier Scientific D10056) for 48 h prior to cytotoxic drug treatment.

### Cell proliferation assays

To assess clonogenic survival after drug treatments, 1 × 10^3^ cells were seeded in 96-well white flat bottom plates (Costar 3917) and treated with cytotoxic drugs for 24 or 48 h. Following drug removal, cells were washed, refed with fresh medium and allowed to recover for 120 h. Cell numbers were determined by alamarBlue fluorimetric assays^73^. Graphpad Prism 5 was used for curve fitting and statistical analysis. The IC_50_ refers to the drug concentration at which cell number in these assays was reduced by 50% relative to no-treatment control; all values were calculated using nonlinear regression for triplicate samples. To visualize cell outgrowth in a 12-well plate format, 3 × 10^4^ cells were seeded per well, treated as above and stained with crystal violet; all assays were repeated at least twice. The number of cells seeded per well was chosen so that after the recovery period, viable cells would normally form a nearly confluent monolayer.

### Ribosome biogenesis analysis

Hybridization analysis of rRNA was done according to the previously described protocols^74^.^32^P (orthophosphate) labeling of rRNA was done as described in^75^.

### Analysis of DNA damage markers

Cells were seeded into 6-well plates (4.5 × 10^5^ cells per well) and treated with 0.4 μM CPT for 1 h to induce DNA damage. For γH2AX analysis, cells were washed in 1 ml of PBS twice and lysed in 300 μl NP40 buffer (50 mM Tris-HCl pH 8, 150 mM NaCl, 50 mM NaF, 1 mM EDTA, 1 mM EGTA, 0.5% NP40) by slowly rocking the plate for 20 min at 4 °C. Lysed cells were scraped into microcentrifuge tubes, centrifuged at 8,500 rpm (6,800 × g) for 5 min at 4 °C, pellets were resuspended in 40 μl 0.1 M HCl and histones were extracted by incubation at room temperature for 15 min followed by centrifugation as above. LDS sample buffer (Thermo Fisher B0007) was added to the supernatants for storage and neutralization of HCl. Samples were analyzed by electrophoresis on 16% SDS-PAGE gels, followed by immunoblotting. Immunofluorescence analysis was done with anti-phospho-ATM monoclonal antibodies and Alexa Fluor 647-congugated anti-mouse antibodies. Nuclei were counterstained with Hoechst 33342. Images were acquired with a Zeiss Axio Observer microscope using Apotome optical sectioning and processed with the Zeiss ZEN Blue software. Quantification of the Alexa/Hoechst signal ratio in individual nuclei was done using Fiji.

### Cell cycle analysis

4.5 × 10^5^ cells (per well) were seeded into 6-well plates, incubated with or without 1 mM IPTG for 24 h and treated with 0 or 0.4 μM CPT for 24 h. Following drug treatment, cells were trypsinized, pelleted at 1,500 rpm (200 × g) for 5 min, resuspended in PBS containing 0.5% BSA, 0.2% NP40, 100 μg/ml RNase A (Amresco) and 25 μg/ml propidium iodide. For Rps19-depleted cells that contain RFP in their shRNA cassette, cells were instead fixed with 70% ethanol and stained in 50 mM Tris-HCl (pH 7.4), 0.5% BSA, 100 μg/ml RNase A and 35 nM SYTOX Green (Thermo Fisher). Samples were incubated for 30 min with gentle rocking in the dark at 37 °C and analyzed on an Attune Acoustic Focusing Cytometer with subsequent data analysis in FlowJo version 10. All cell cycle analyses were performed at least twice.

### CRISPR/Cas9 generation of p53-null cells

Guide RNAs were designed using CRISPR design tools at e-crisp.org and crispr.mit.edu. The corresponding oligonucleotides were cloned into the Bbs I restriction sites of eSpCas9(1.1), which carries a Cas9 variant with enhanced specificity (ref. 76; Addgene plasmid #71814). 1.5 × 10^5^ cells per well were seeded into a 12-well plate and transfected with 0.2 μg pPGK-puro and 0.9 μg guide RNA constructs using Polyfect (Qiagen). After 24 h, cells were split into 10-cm dishes and 2.5 μg/ml puromycin was added for 48 h to select transfected cells. Single-cell clones were isolated from the transfected cell pools by limiting dilution. To analyze genomic sites targeted by CRISPR, genomic DNA was isolated from 3 × 10^4^ cells by lysis in 50 μl of 10 mM Tris-HCl (pH 8), 1 mM EDTA, 0.1% SDS, 60 μg proteinase K (Thermo Fisher EO0491) for 1 h at 55 °C in an Eppendorf Thermomixer at 500 rpm. Proteinase K was inactivated at 95 °C for 10 min, samples were centrifuged at 13,000 rpm (15,900 × g) for 3 min at 4 °C to remove cell debris, 2 μl of supernatants were amplified in 20 μl PCR reactions using DreamTaq (Thermo Fisher) and cloned into pGEM-T Easy (Promega) for sequencing. For protein analysis, p53 was induced with 10 μM 9-aminoacridine (Sigma) for 6 h^77^, cells were washed in PBS twice and lysed in NP40 buffer containing 1 ×SIGMAFAST protease inhibitors with an additional 1 mM AEBSF and 0.2 mM sodium orthovanadate by rocking the plate for 20 min at 4 °C.

### Differential killing of cells

D411 (p53+) and D411/C5 (p53−) cells were transfected with pCMV-IE-tdTomato (gift of Dr. H. Houbaviy) and pGreen Lantern, respectively, using a calcium phosphate transfection procedure, selected with puromycin, and single stably transfected cell clones were then isolated by limiting dilution. In flow cytometry analysis, gates were set to include less than 0.5% non-fluorescent cells in the quadrants for each fluorescent label. For CPT treatment, D411/RT and D411/C5/GL cells (7.5 × 10^4^ each) were seeded together in a 6-well plate, incubated with 1 mM IPTG for 24 h and then treated or not with 0.4 μM CPT for 24 h, a dose that kills proliferating cells. Following recovery in drug-free media for 24 h, cells were trypsinized and transferred to a new 6-well plate for an additional 36 h, harvested with trypsin, resuspended in 1 ml PBS and analyzed by flow cytometry.

### Data Availability

No datasets were generated or analysed during the current study.

## Electronic supplementary material

Supplementary Information
