# Supplementary material for: Inhibition of post-transcriptional steps in ribosome biogenesis confers cytoprotection against chemotherapeutic agents in a p53-dependent manner
Source: Sci Rep. 2017 Aug 22;7:9041. doi: 10.1038/s41598-017-09002-w (PMC5567254; doi:10.1038/s41598-017-09002-w)
Supplement: Supplementary file 1 — Supplementary Information [file 41598_2017_9002_MOESM1_ESM.pdf]

Supplementary Information

Inhibition of post-transcriptional steps in ribosome biogenesis confers cytoprotection against chemotherapeutic agents in a p53-dependent manner

Russell Sapio, Anastasiya N. Nezdyur, Matthew Krevetski, Leonid Anikin, Vincent J. Manna, Natalie Minkovsky, and Dimitri G. Pestov

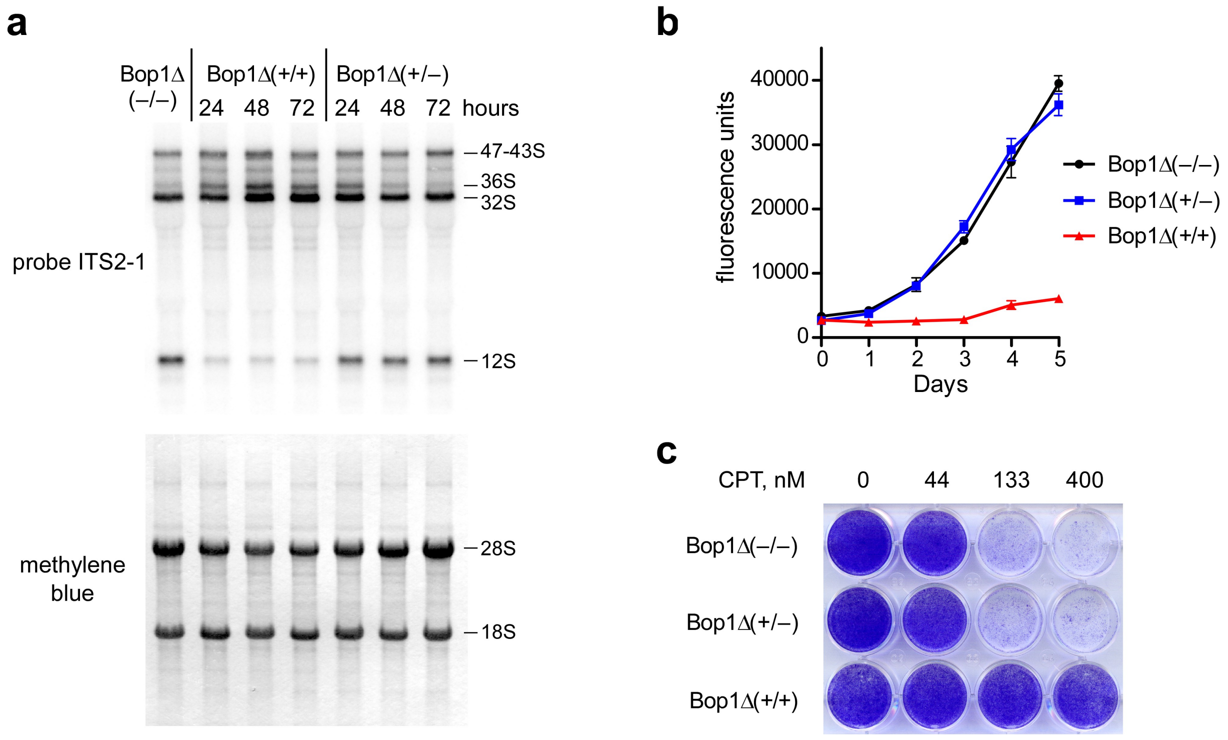

Supplementary Figure S1. Bop1Δ phenotype is reversible. D411 cells harboring IPTG-inducible Bop1Δ were maintained without IPTG (–/–), treated with IPTG (+/+) or incubated with IPTG for 24 h and then switched to medium without IPTG (+/–) for the indicated periods of time. (**a**) Impaired formation of 12S pre-rRNA and accumulation of 36S pre-rRNA are observed after Bop1Δ induction, as previously described[1]; these effects on pre-rRNA maturation are reversed by removal of IPTG. Northern hybridization was done with a probe specific for the ITS2 region in pre-rRNA[2]. The same membrane was stained with methylene blue to control loading. (**b**) Proliferation rate in D411 cells incubated with IPTG for 24 h and then released into normal medium is similar to untreated cells. Relative cell numbers were determined using the CyQUANT cell proliferation assay (Thermo Fisher) in 96-well plates using the manufacturer's protocol that incorporates RNase treatment to eliminate variations due to changes in the cellular RNA content. Data points show mean values and s.e.m. in 5 replicates. (**c**) Drug resistance is lost when Bop1Δ is shut down. Cells were treated with CPT as in Figure 1c. In the (+/–) culture, CPT treatment was started 24 h after the removal of IPTG.

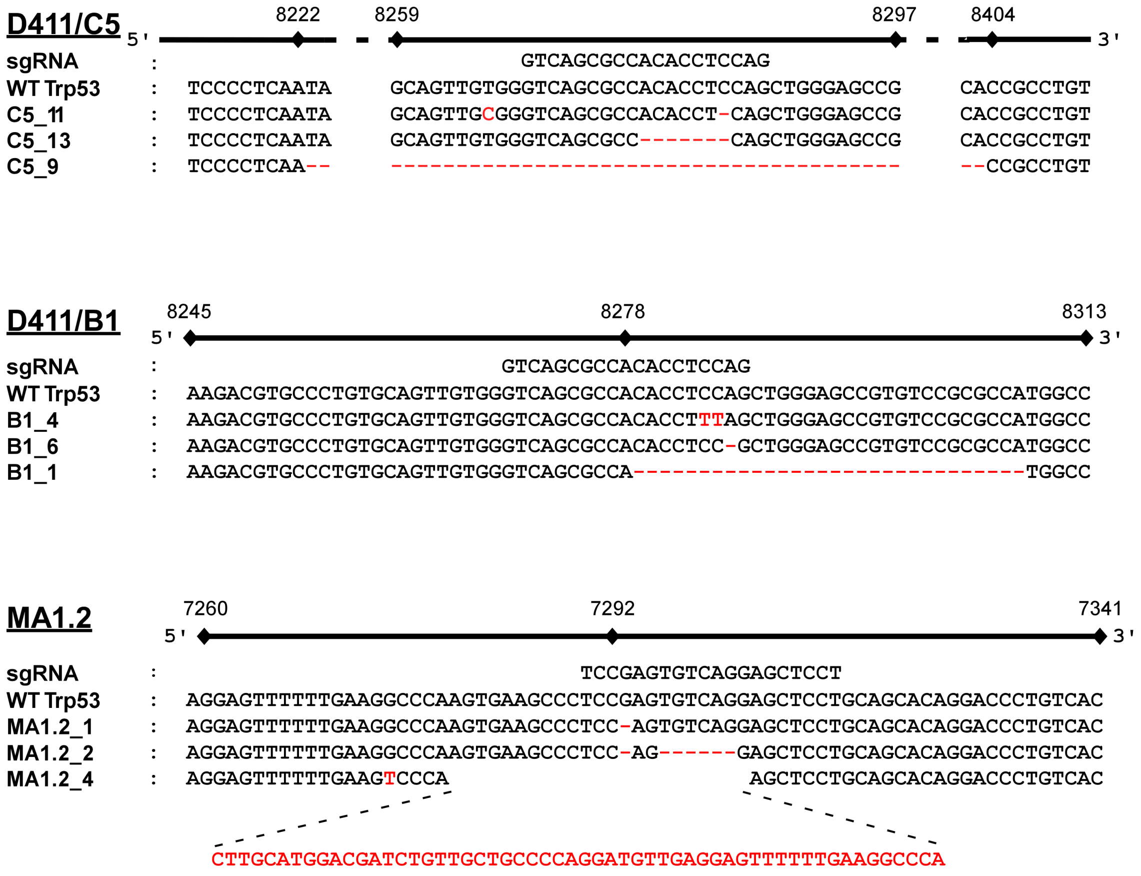

Supplementary Figure S2. Generation of p53-null mouse cells via CRISPR/Cas9-mediated gene editing. We used an sgRNA to target exon 4 of the *Trp53* gene in Bop1Δ-inducible D411 cells. To address the possibility of off-target effects that might be particular to that guide, we used a different sgRNA that targeted exon 3 of *Trp53* when performing knockout in 3T3 cells. In the generated cell lines D411/C5, D411/B1 and 3T3/MA1.2, all of which lacked detectable p53 protein based on western blot analysis, we PCR amplified the *Trp53* sequence around the targeted sites, cloned the PCR products into pGEM-T Easy (Promega) and sequenced multiple plasmids. Representative sequences are shown, with indels indicated in red. Also shown are sgRNAs used in each case and the corresponding portions of the wild-type *Trp53* sequence. We observed three unique editing events in each tested cell line, suggesting that the parental D411 and 3T3 cells (which are otherwise mostly tetraploid) carried three alleles of *Trp53.*

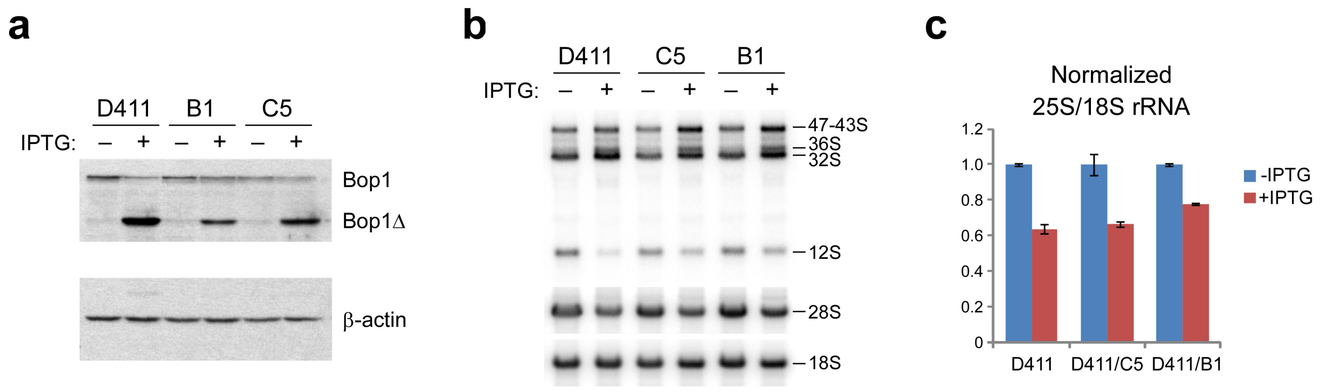

Supplementary Figure S3. Additional characterization of D411 cells and its p53-deficient derivatives D411/C5 and D411/B1. (**a**) Immunoblotting analysis of Bop1Δ expression 24 h after induction with IPTG using antibodies raised against mouse Bop1[3]. The membrane was reprobed with β-actin antibodies to control loading. (**b**) Hybridization analysis using pre-rRNA probe ITS2-1[2] and probes against mature 28S and 18S rRNA. Bop1Δ was induced with IPTG for 48 h. (**c**) Quantification of the 28S/18S rRNA ratios shows reduced 28S rRNA synthesis in D411 cells and its p53-null derivatives after 48 h induction of Bop1Δ with IPTG. Graphs show mean values normalized to the mean ratio in uninduced cells, error bars show range in two biological replicates.

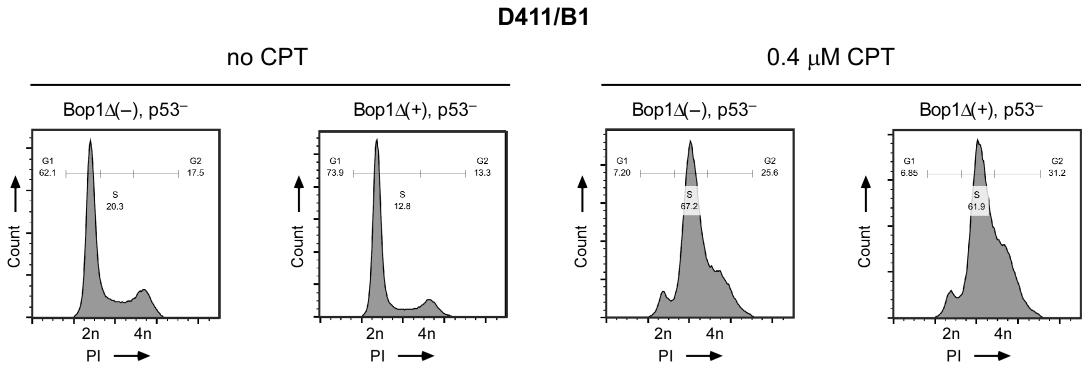

Supplementary Figure S4. Representative cell cycle distribution profiles of the p53-null D411/B1 cell line. Cells were either incubated or not with IPTG for 24 h to induce Bop1Δ. Where indicated, cells were exposed to 0.4 μM CPT for an additional 24 h. After drug treatment, cells were harvested, permeabilized and their cell cycle profiles determined by staining with propidium iodide (PI).

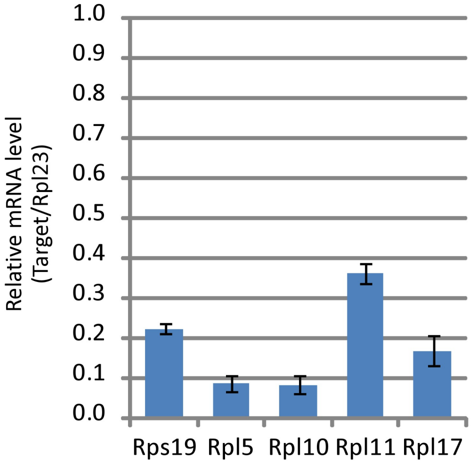

Supplementary Figure S5. shRNA knockdown efficiencies. Ratios of the shRNA-targeted ribosomal proteins mRNAs to that of the reference ribosomal protein Rpl23 were determined by quantitative RT-PCR as previously described[4]. Target/Rpl23 mRNA ratios were measured 72 h after induction of shRNAs with doxycycline and normalized to the average ratio in cells cultured without doxycycline. Data are mean values in technical replicates; error bars, s.e.m; *n* = 3-4.

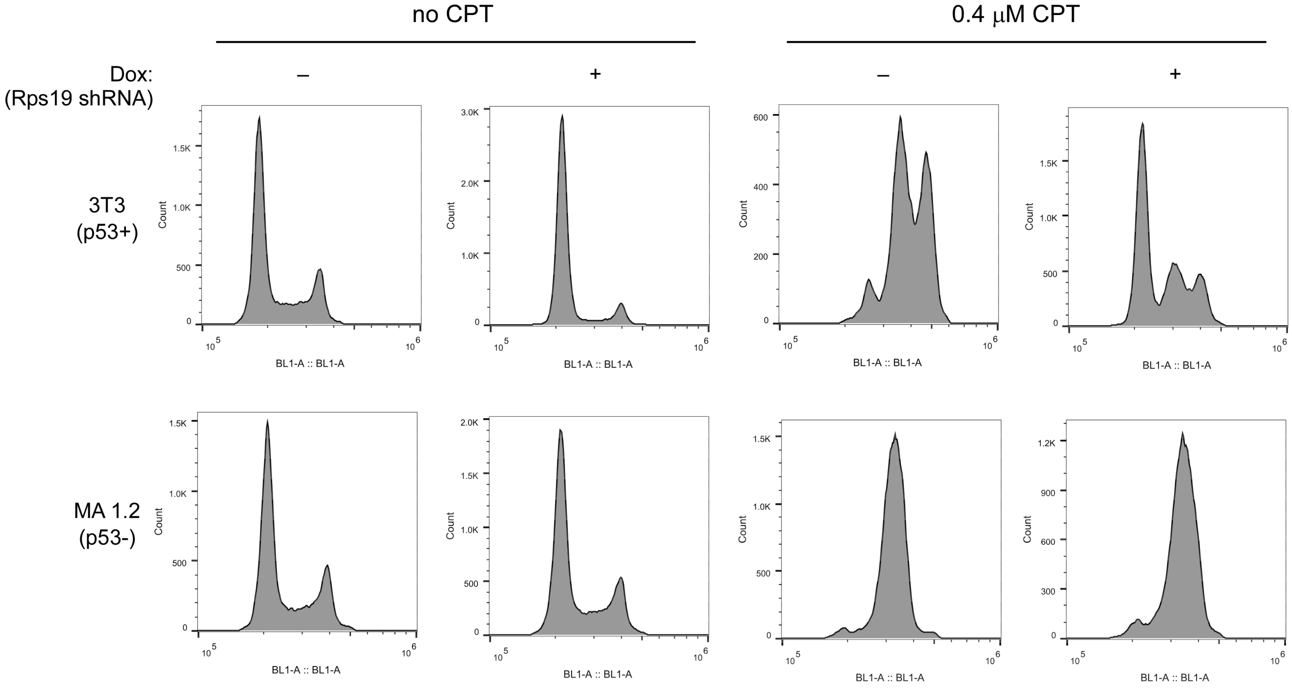

Supplementary Figure S6. Cell cycle analysis in Rps19 shRNA cell lines. Rps19 shRNA was induced for 48 h with Dox or not induced in 3T3 cells and their p53-null derivative MA1.2. Where indicated, cells were treated with CPT for 24 h. Cell cycle profiles were determined in ethanol-fixed cells by staining DNA with SYTOX Green.

Figure 1e

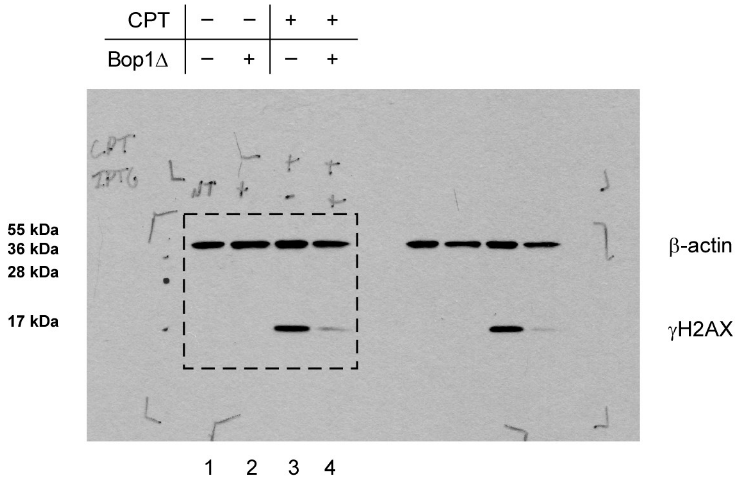

Figure 3a

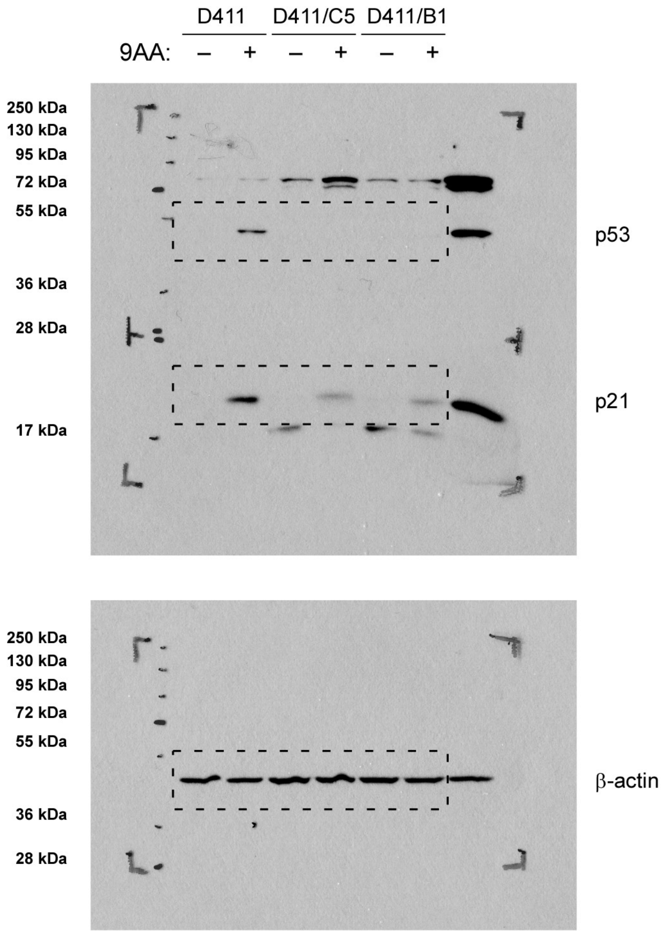

Supplementary Figure S7. Uncropped images of the immunoblots.

Supplementary References

1. Strezoska, Z., Pestov, D. G. & Lau, L. F. Functional inactivation of the mouse nucleolar protein Bop1 inhibits multiple steps in pre-rRNA processing and blocks cell cycle progression. *J. Biol. Chem.* **277,** 29617–29625 (2002).
2. Lapik, Y. R., Fernandes, C. J., Lau, L. F. & Pestov, D. G. Physical and functional interaction between Pes1 and Bop1 in mammalian ribosome biogenesis. *Mol. Cell* **15,** 17–29 (2004).
3. Strezoska, Z., Pestov, D. G. & Lau, L. F. Bop1 is a mouse WD40 repeat nucleolar protein involved in 28S and 5.8S rRNA processing and 60S ribosome biogenesis. *Mol. Cell. Biol.* **20,** 5516–5528 (2000).
4. Wang, M., Anikin, L. & Pestov, D. G. Two orthogonal cleavages separate subunit RNAs in mouse ribosome biogenesis. *Nucleic Acids Res.* **42,** 11180–11191 (2014).
